# A novel modified-curcumin 2.24 resolves inflammation by promoting M2 macrophage polarization

**DOI:** 10.1038/s41598-023-42848-x

**Published:** 2023-09-19

**Authors:** Jie Deng, Lorne M. Golub, Hsi-Ming Lee, Heta-Dinesh Bhatt, Francis Johnson, Tian-min Xu, Ying Gu

**Affiliations:** 1 Department of Orthodontics, Peking University School and Hospital of Stomatology & National Clinical Research Center for Oral Diseases & National Engineering Laboratory for Digital and Material Technology of Stomatology & Beijing Key Laboratory of Digital Stomatology, 22 Zhongguancun South Avenue, Haidian District, Beijing, 100081 People’s Republic of China; 2 Department of Orthodontics, Nanjing Stomatological Hospital, Affiliated Hospital of Medical School, Nanjing University, Nanjing, People’s Republic of China; 3 Department of Oral Biology and Pathology, School of Dental Medicine, Stony Brook University, Stony Brook, NY 11794 USA; 4 Department of Chemistry and Pharmacological Sciences, School of Medicine, Stony Brook University, Stony Brook, NY 11794 USA; 5 Department of General Dentistry, School of Dental Medicine, Stony Brook University, Stony Brook, NY 11794 USA

**Keywords:** Dental diseases, Pharmaceutics, Inflammation, Diabetes

## Abstract

To assess resolving-like activity by a novel chemically-modified curcumin (CMC2.24) in a “two-hit” model of diabetes-associated periodontitis. Macrophages from rats were cultured in the presence/absence of either *Lipopolysaccharide* (LPS, 1st hit); or advanced-glycation-end products (AGE, 2nd hit); or both combined. CMC2.24 was added as treatment. The conditioned media were analyzed for MMP-9, cytokines (IL-1β, IL-6, TNF-α), resolvins (RvD_1_, RvE_1_, lipoxin A_4_), and soluble receptor for AGE (sRAGE). The phenotypes of M1/M2 macrophage were analyzed by flow cytometry. Both LPS/AGE-alone, and two-combined, dramatically increased the secretion of MMP-9 by macrophages. CMC2.24 “normalized” the elevated levels of MMP-9 under all conditions. Moreover, CMC2.24 significantly reduced the secretion of IL-1β and IL-6 with a fewer effects on TNF-α. Importantly, CMC2.24 increased RvD_1_ and sRAGE secretion by macrophages exposed to LPS/AGE; and both treatment groups exhibited increased M2 relative to M1 populations. Furthermore, scatter-diagram showed the macrophages gradually shifted from M1 towards M2 with CMC2.24-treated, whereas LPS/AGE-alone groups remained unchanged. CMC2.24 “normalized” cytokines and MMP-9, but also enhanced RvD_1_ and sRAGE in macrophages. Crucially, CMC2.24 appears to be a potent inhibitor of the pro-inflammatory M1 phenotype; and a promotor of the pro-resolving M2 phenotype, thus acting like a crucial “switch” to reduce inflammation.

## Introduction

The connection between diabetes and periodontitis has received constant attention in recent years. Both involve a broad range of inflammatory/collagenolytic cascades. Diabetes is known to promote unusually severe periodontitis when not optimally controlled^1, 2^, whereas periodontitis has an exuberant “host-response” in the periodontium to generate numerous inflammatory mediators and proteinases. They not only break the collagen-rich deeper periodontal tissues, thus leading to tooth loss; but also cause insulin resistance^3, 4^. How can disease, in one part of the body transmit signals to the system, or vice versa, affecting each other?

Recently, a “two-hit model” was proposed by Golub et al.^5^, to demonstrate a bi-directional manner between diabetes and periodontitis. The “1st hit” is caused by microbial biofilm (dental plaque), which acts as a reservoir of periodontal pathogens. The binding of lipopolysaccharide (LPS) or endotoxin from the pathogens to toll-like receptor 2/4 in macrophages (Mφs) or polymorphonuclear leukocytes (PMNs) triggers an inflammatory response. This response leads to the production of excessive pro-inflammatory mediators and proteinases in the periodontium^6^, which include cytokines [e.g., interleukin-1 beta and 6 (IL-1β, IL-6), tumor necrosis factor-alpha (TNF-α)]; and matrix metalloproteinases (i.e., MMP-2 and 9)^7^. They contribute to the destruction of both soft and hard periodontal tissues. Additionally, these mediators can be carried through circulation to the liver, resulting in the release of additional inflammatory mediators systemically. These systemic mediators, including C-reactive protein, IL-6, prostaglandin E_2_, as well as other MMPs, affect overall systemic conditions and might also exacerbate periodontal breakdown. Moreover, diabetes acts as the “2nd hit” during this interaction^5^. If uncontrolled hyperglycemia exists, advanced-glycation-end products (AGE) are formed to react with their cellular receptors for AGE (RAGE) on the surfaces of macrophages or other inflammatory cells. It leads to adverse events including elevated oxidative stress (i.e., reactive oxygen species); and osmotic stress (causing raised production of proinflammatory cytokines and MMPs). Eventually, these events amplify the inflammatory cascades and exacerbate periodontitis through the circulation as well^8, 9^. However, the soluble receptor for AGE (sRAGE) counteracts the adverse effects of AGE-RAGE interaction by competing with RAGE for binding with AGE. Low levels of sRAGE have been proposed as early predictors of Type II diabetes^10^.

At the cellular level, Mφs exhibit extreme heterogeneity both in normal and pathological conditions due to a major feature of “plasticity.” In this regard, Mφs are generally categorized into two broad subsets as either classically activated M1 (pro-inflammatory) or alternatively activated M2 (anti-inflammatory/pro-resolving) phenotypes, indicating their dual role as “killers” (M1) and/or “builders” (M2)^11, 12^. During chronic inflammation (diabetes and/or periodontitis), relevant cellular and molecular mechanisms drive Mφs in these pathological conditions to become M1 pro-inflammatory phenotypes^11–13^. In contrast, processes that promote the conversion of M1 to the M2 phenotypes help resolve inflammation, inducing tissue regeneration rather than tissue destruction^14^.

The emergence of host modulation therapy (HMT), as a targeted treatment strategy, accelerates the development of novel drugs^15^. Interestingly, recent studies^3, 4, 16^ indicated that treating diabetic people with an MMP inhibitor, doxycycline, resulted in decreased inflammation and increased insulin sensitivity as well as improved glucose control^3^. Other examples of pleiotropic MMP-inhibitor drugs, notably the non-antimicrobial formulations of tetracycline compounds can reduce the severity of several systemic diseases (e.g., rheumatoid arthritis, post-menopausal osteoporosis), as well as periodontitis, in humans^17–21^.

HMT, in part, is to restore the balance between (a) pro-inflammatory mediators and anti-inflammatory mediators; and, (b) destructive enzymes and enzyme inhibitors^15^. Regarding resolvins and lipoxins, both are pro-resolving lipid mediators derived from omega-3 (or n-3) polyunsaturated fatty acids^22^. As one category of HMT agents, major resolvins (RvD_1_ and RvE_1_) and Lipoxin A_4_ (LxA_4_) have a fundamental role in resolving inflammation^23–25^. In contrast to anti-inflammatory drugs (e.g., nonsteroidal anti-inflammatory drugs and corticosteroids), resolvins and lipoxins do not suppress acute inflammation, which is necessary to combat infectious agents, but prevents this acute inflammatory response from being prolonged which, would be tissue-damaging^26^.

To date, the only HMT agent for treating patients with periodontitis that has been governmentally approved is Periostat® (doxycycline hyclate) developed by our group^27^. And recently, we further developed a novel chemically-modified curcumin 2.24 (CMC2.24) (a triketonic phenylaminocarbonyl curcumin). As one of the most effective and newer HMT agents, CMC2.24 is a “lead” compound. It was found to be safer and most effective based on in vitro, cell and tissue culture, and in vivo studies on rat and dog models of experimental periodontitis and diabetes^28–36^. In this current study, we describe the additional mechanisms which were not previously identified. The objective is to expand on and discover additional host modulatory activities of this pleiotropic CMC2.24, using a “two-hit” Mφ cell-culture model of diabetes-associated periodontitis. We hope that CMC2.24 can be a beneficial and novel therapeutic HMT agent in the management of chronic inflammatory diseases, including diabetes and periodontitis.

## Material and methods

### Animals (source of monocytes/macrophages) and ethical approval

Sprague–Dawley (SD) rats (Body Weight/Age: 301–325 g/66–71 days) were purchased from Charles River (Stone Ridge, NY, USA). All rats were housed in the Division of Laboratory Animal Resources (DLAR) at Stony Brook University, with care provided by the center’s personnel. All procedures were conducted at the same location. DLAR is an AAALAC International accredited facility; and follows the Animal Welfare Act (USDA enforced), the Public Health Service Act (OLAW enforced), and NY State law (DOH enforced). Protocols for animal studies were approved by Stony Brook University’s Institutional Animal Care and Use Committee (IACUC# 230,617–23). Sixteen male adult SD rats were used to collect peritoneal and blood-derived Mφs. All experiment methods were carried out in accordance with relevant guidelines and complied with ARRIVE 2.0 guidelines^37^.

### Chemical reagents

CMC 2.24 was synthesized and provided by Chem-Master Intl. Inc. (99.5% pure, Stony Brook, NY, USA). All cell culture reagents and other chemical reagents were purchased from Thermo Fisher Scientific (Waltham, MA, USA).

### Cell culture studies

#### Peritoneal Mφs

Mφs were obtained from peritoneal washes (PW) of 8 SD rats. Briefly, 15 mL of cold sterile PBS (pH7.4) containing 3 mM of ethylenediaminetetraacetic acid (EDTA) intraperitoneally (i.p.) was injected into the peritoneal cavity, and the peritoneum was gently massaged for 1 min. Approximately 12 mL/per rat of PW was collected and stored on ice (4 °C). These lavages were centrifuged (1000 rpm, 4 °C, 5 min) to separate supernatant cell-free peritoneal fractions (CFPFs) and resident mixed cells that were re-suspended in 25 mL/each sample of PBS/3 mM EDTA. The cell suspension was layered onto Lymphoprep™ (Accurate Chemical & Scientific Corporation, Westbury, NY, USA) at a ratio of 2 to 1–1.5 (v/v) and centrifuged at 1,800 rpm for 30 min at 25 °C. The resident peritoneal Mφs were isolated and purified from the mixed cells by density gradient centrifugation and adherence as described previously^38^. The peritoneal Mφs were cultured in macrophage serum-free media (SFM) (Thermo Fisher Scientific Inc., Boston, MA, USA) in 24-well plates. Each well contained 10^6^ cells/mL, supplemented with 100 units/mL penicillin, and 100 μg/mL streptomycin, in a humidified atmosphere of 5% CO_2_ and 95% air at 37 °C.

**Figure 1:**
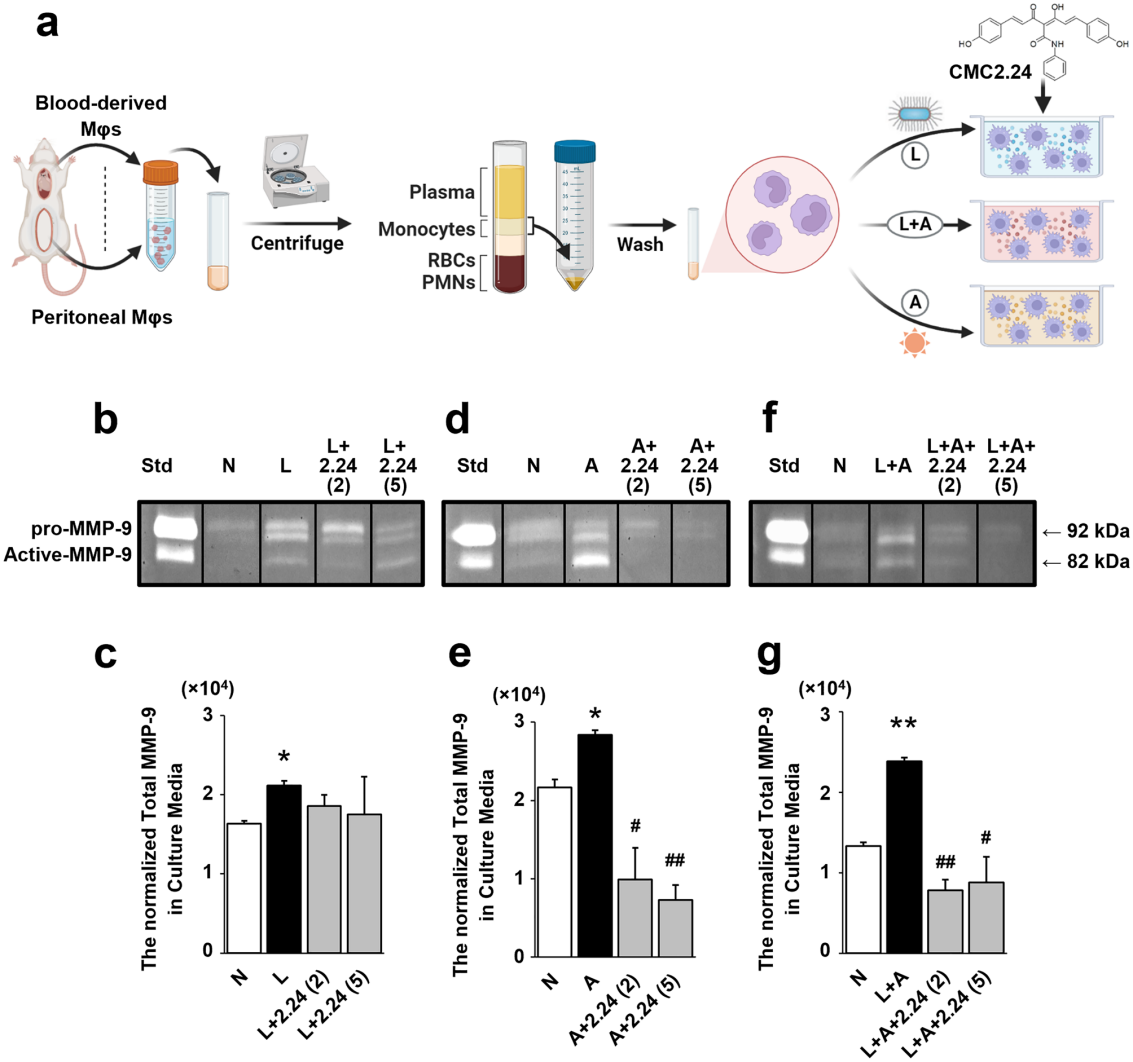
The study design of a “two-hit” cell-culture model of diabetes-associated periodontitis. (**a**) Resident peritoneal and blood-derived Mφs were harvested from the rat, and separated by density gradient centrifugation, respectively. In an *ex-vivo* cell culture, Mφs were incubated in the absence (normal control), or presence of either LPS (L, the “1st hit”); or AGE (A, “2nd hit”); or both combined (L + A). CMC2.24 was added to the cultures at 2 or 5 μM. MMP-9 activity was measured by gelatin zymography and scanned densitometrically. Effects of CMC2.24 on MMP-9 in the peritoneal Mφs challenged by LPS-alone (**b**,**c**), AGE-alone (**d**,**e**), or the “two-hit” (**f**,**g**). N, the normal group without any challenge or treatment. L, the LPS-alone challenged group. L + 2.24 (2 or 5), the low (2 μM) and high (5 μM) concentrations of CMC2.24-treated groups. A, the AGE-alone challenged group. A + 2.24 (2 or 5), the low (2 μM) and high (5 μM) concentrations of CMC2.24-treated groups. L + A, the “two-hit” challenged group. L + A + 2.24 (2 or 5), the low (2 μM) and high (5 μM) concentrations of CMC2.24-treated groups. Each value represents the mean (n = 3/group) ± the standard error. *p < 0.05, **p < 0.005, values were compared between N and L/A/(L + A)-challenged groups at 18-h. ^#^p < 0.05, ^##^p < 0.005, values were compared between L/A/(L + A)-challenged and L/A/(L + A) + 2.24 (2 or 5)-treated groups at 18-h. The grouping of gels were cropped from different parts of the same gel. The full-length gels were included in the Supplementary Information files (S1-4.)

The Mφs were distributed into 10 groups (triplicates/group) to mimic the “two-hit” model. The “1st hit” involved the absence (Normal control, N) or presence of *P.g.* LPS (L) at a final concentration of 100 ng/mL; the “2nd hit” involved the absence (N) or presence of AGE (A) at a final concentration of 100 µg/mL; or the “two hits” combined (L + A). CMC2.24 was added to the cultures at final concentrations of 2 or 5 μM [L + 2.24 (2 or 5); A + 2.24 (2 or 5); L + A + 2.24 (2 or 5)]. (Fig. 1a). *P.g.* LPS was purchased from Invitrogen (San Diego, CA, USA). AGE-BSA-II (glycated Bovine Serum Albumin) was purchased from BioVision, Inc. (Milpitas, CA, USA).

After 18-h incubation, the conditioned media were collected and analyzed for pro- (92 kDa) and active- (82 kDa) MMP-9 by gelatin zymography, and for cytokines (IL-1β, IL-6, and TNF-α), sRAGE, and resolvins (RvD_1_, RvE_1_, and LxA_4_), all by enzyme-linked immunosorbent assay (ELISA).

#### Blood-derived Mφs

Resident blood-derived Mφs were isolated from the heart of 8 SD rats by cardiac puncture. In brief, 10–15 mL of whole blood from each rat was drawn, then transferred to the heparin-coated tubes (anti-clotting). Mφs isolation was achieved by the same procedure as described above (Fig. 1a), then cultured in the Teflon Beakers (Savillex, MN, USA) to prevent cell attachment (no adherence)^39, 40^. At time 0, Mφs were isolated and distributed into 6 groups (triplicates/group) to mimic the “two-hit” model of diabetes-associated periodontitis: in the absence (N), or presence of either LPS (L) or AGE (A). CMC2.24 was added to the cultures of different groups at a final concentration of 5 μM (N + 2.24, L + 2.24, and A + 2.24), then harvested at 0, 6, and 18 h for flow cytometry analysis.

### Flow cytometry

At 0, 6, and 18 h, Mφs were harvested and immediately labeled by fluorescence-conjugated antibodies against different cell surface markers to identify M0, M1, and M2 phenotypes. The CD11b/c^+^ (1:100 dilution, BioLegend Inc., San Diego, CA, USA) was used as a pan-macrophage marker to gate the total populations of macrophage, including M0 phenotype and the nonpolarized resting Mφs^41–44^. The CD38^+^ (1:100 dilution, BioLegend Inc., San Diego, CA, USA) was used for labeling M1 phenotype (CD11b/c^+^ CD38^+^)^44, 45^; and the CD163^+^ (1:200 dilution, BioRad Laboratories Inc., Hercules, CA, USA) was used for labeling M2 phenotype (CD11b/c^+^ CD163^+^)^46–48^. Flow cytometry analysis was using FACSCalibur™ (BD Biosciences, San Jose, CA, USA). The M2/M1 ratio was calculated by using the percentage of gating for the M2 phenotype divided by the M1 phenotype at 0, 6, and 18 h, respectively^49^.

### Gelatin zymography for MMP-9 analysis

After 18-h incubation, the conditioned media from cultured peritoneal Mφ were collected and analyzed for pro- (92 kDa) and active- (82 kDa) MMP-9 by gelatin zymography as described previously^32, 33, 50, 51^. The clear zones of lysis against a background indicated gelatinolytic activity and were scanned densitometrically with Invitrogen™ iBright™ FL1000 Imaging Systems (Thermo Fisher Scientific, Inc., Boston, MA, USA). Results were analyzed by Image J to quantitatively assess gelatinase activity^31^. MMP-9 standard was purchased from R&D Systems, Inc. (Minneapolis, MN, USA).

### ELISA

Quantikine® ELISA kits for IL-1β (RLB00, SRLB00, PRLB00), IL-6 (R6000B, SR6000B, PR6000B), and TNF-α (RTA00, SRTA00, PRTA00) were purchased from R&D Systems, Inc. (Minneapolis, MN, USA). RvD_1_ (Cat# 500,380) was purchased from Cayman Chemical. (Ann Arbor, Michigan, USA). RvE_1_ (Cat# MBS2604861) and soluble receptor of AGE (sRAGE) (Cat# MBS722302) were purchased from MyBioSource, Inc. (San Diego, CA, USA), and LxA_4_ (Cat# 407,010) was purchased from Neogen Corporation (Lexington, Kentucky, USA). All measurements were performed according to the kit manufacturer’s instructions.

### Statistical analysis

SPSS21.0 (Statistical Package for Social Sciences, IBM) was used to analyze the data. The levels of MMP-9, pro-inflammatory cytokines, resolvins, sRAGE, and the percentage of different Mφ populations were determined using analysis of variance (*ANOVA*), and also using Student’s *t*-test (two investigators carried out the data analysis, separately), with p < 0.05 taken as statistically significant. Each value represented the mean (n = 3/group) ± the standard error of the mean (S.E.M.). All experiments were independently performed at least three times.

## Results

### Effects of CMC2.24 on MMP-9

Gelatin zymography analysis showed that both LPS (the “1st hit”) and AGE-alone (the “2nd hit”) challenged groups significantly increased total-MMP-9 levels by 20.9% and 20.1% (p < 0.05; p < 0.05), compared to the N group (Fig. 1b–e). which reflected both elevated pro- and active-forms of this MMP (Fig. 1b,d). A trend towards reduction in the total-MMP-9 levels towards normal was seen in both low (2 μM) and high (5 μM) concentrations of CMC2.24-treated groups, although both concentrations of CMC2.24 treatment were not statistically significant (p > 0.05) compared to the L-alone (Fig. 1c). Importantly, the total-MMP-9 levels in these CMC2.24-treated groups were not different from those in the N group (p > 0.05), indicating the “normalizing effect” of CMC2.24. In addition, treatment with 2 and 5 µM of CMC2.24 did significantly reduce the total-MMP-9 levels by 65.1% and 74.4% (p < 0.05; p < 0.005), respectively, compared to the A-alone (Fig. 1e). Also, there appeared to be a dose–response effect in the CMC2.24-treated groups since the higher concentration (5 µM) of CMC2.24 produced a greater reduction in the total-MMP-9 levels (Fig. 1e).

When the two-hit system (L + A) was used, it dramatically increased the total-MMP-9 levels by 42.1% (p < 0.005) compared to the N group (Fig. 1f–g). It appeared to reflect elevated pro- and active-forms of this MMP as well (Fig. 1f). Treatment with 2 and 5 µM of CMC2.24 largely reduced total-MMP-9 levels by 67.2% and 63.1% (p < 0.005; p < 0.05), respectively, compared to the L + A group (Fig. 1g).

### Effects of CMC2.24 on pro-inflammatory cytokines

**Figure 2:**
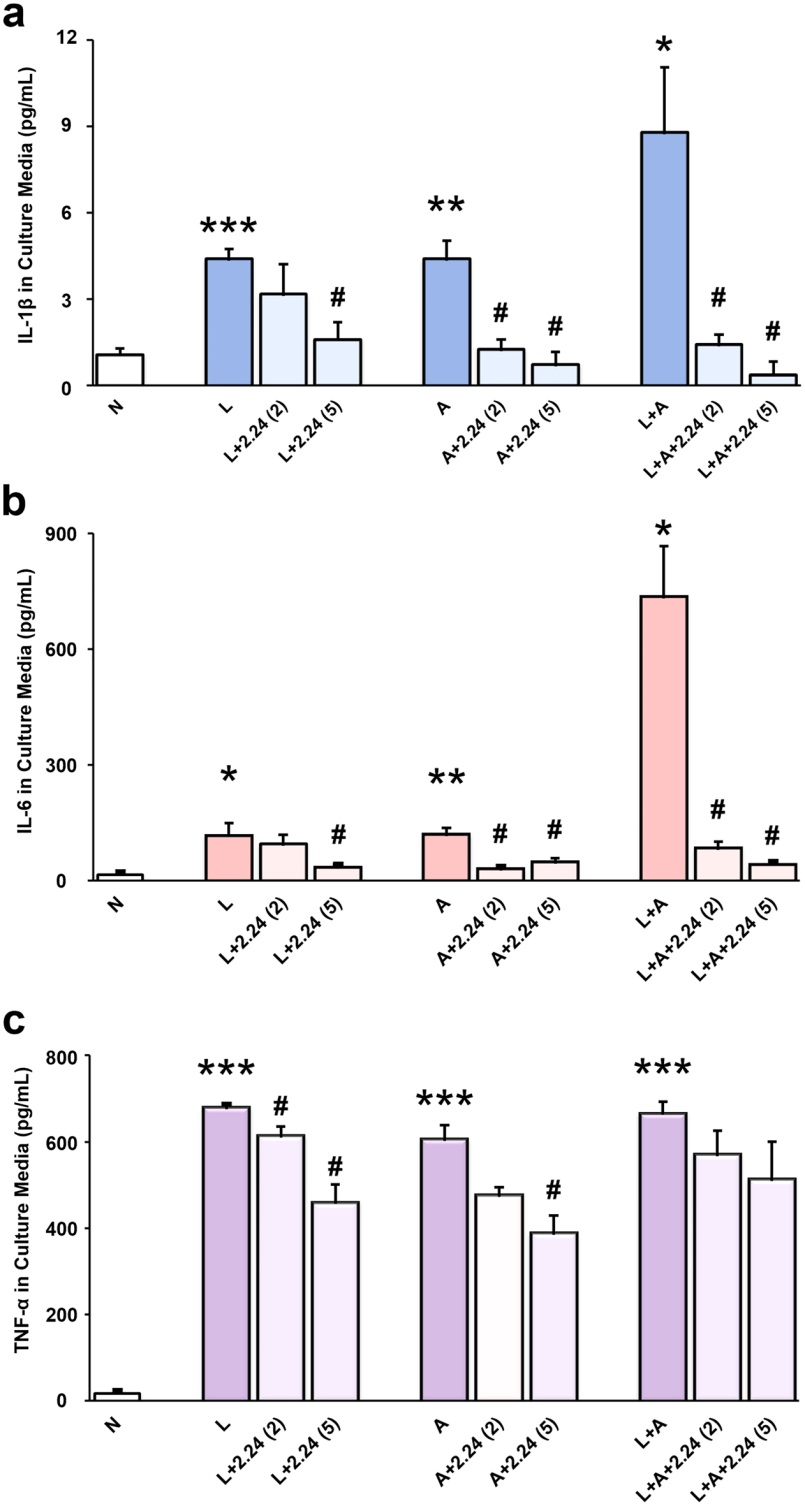
Effects of CMC2.24 on pro-inflammatory cytokines. The effects of CMC2.24 on the concentrations (pg/mL) of IL-1β (**a**), IL-6 (**b**) and TNF-α (**c**) in rat peritoneal Mφs challenged by LPS-alone, AGE-alone, or the “two-hit”, respectively; and treated with different concentrations (2 or 5 μM) of CMC2.24 at 18-h. N, the normal group without any challenge or treatment. L, the LPS-alone challenged group. L + 2.24 (2 or 5), the low (2 μM) and high (5 μM) concentrations of CMC2.24-treated groups. A, the AGE-alone challenged group. A + 2.24 (2 or 5), the low (2 μM) and high (5 μM) concentrations of CMC2.24-treated groups. L + A, the “two-hit” challenged group. L + A + 2.24 (2 or 5), the low (2 μM) and high (5 μM) concentrations of CMC2.24-treated groups. Each value represents the mean (n = 3/group) ± the standard error. *p < 0.05, **p < 0.005, ***p < 0.001, values were compared between N and L/A/(L + A)-challenged at 18-h. ^#^p < 0.05, values were compared between L/A/(L + A)-challenged and L/A/(L + A) + 2.24 (2 or 5)-treated groups at 18-h.

As shown in Fig. 2a, IL-1β concentrations were significantly increased in both L and A-alone (p < 0.001; p < 0.005) challenged groups, and with a synergistic and significant increase in the L + A challenged group (p < 0.05), respectively, compared to the N group. IL-1β concentrations in the L + A group were also significantly greater than each “hit” (L or A) alone (p < 0.05; p < 0.05). When the L-alone was treated with 2 and 5 µM of CMC2.24, the higher concentration (5 µM) of CMC2.24 produced a significant reduction in IL-1β concentrations (p < 0.05), as well as a trend of reduction at the lower concentration (2 µM) of CMC2.24 (p > 0.05). In addition, when the A-alone was treated with 2 and 5 µM of CMC2.24, both concentrations of CMC2.24 treatment significantly reduced IL-1β concentrations (p < 0.05; p < 0.05) in an apparent dose–response manner. Impressively, when the “two-hit” (L + A) group was treated with 2 and 5 µM of CMC2.24, both concentrations of CMC2.24 significantly decreased IL-1β concentrations (p < 0.05; p < 0.05) and brought them back to normal levels in a similar dose–response relationship (Fig. 2a).

In addition, IL-6 concentrations were significantly increased in both L and A-alone (p < 0.05; p < 0.005) groups, also with a similar synergistic and significant increase in the L + A challenged group (p < 0.05), respectively, compared to the N group (Fig. 2b). IL-6 concentrations in the “two-hit” (L + A) group were significantly greater than each “hit”-alone (both p < 0.05). Moreover, the CMC2.24-treated group (5 µM) significantly reduced IL-6 concentrations (p < 0.05), compared to the L-alone. When the A-alone was treated with 2 and 5 µM of CMC2.24, both concentrations significantly reduced IL-6 concentrations (both p < 0.05) compared to the A-alone. Furthermore, when the L + A group was treated with 2 and 5 µM of CMC2.24, both concentrations significantly decreased IL-6 concentrations (both p < 0.05), again, in the apparent dose–response fashion, and brought them back to normal levels.

Next, we examined TNF-α concentrations in the same culture media. As shown in Fig. 2c, TNF-α levels were significantly increased in all three challenged groups: L and A-alone, as well as the L + A groups (all p < 0.001), respectively, compared to the N group. However, the combination of “two-hit” did not increase TNF-α concentrations over the already (apparently) maximum increase in this cytokine produced by each “hit” separately. Impressively, when the L-alone was treated with 2 and 5 µM of CMC2.24, both concentrations significantly reduced TNF-α concentrations (both p < 0.05) in a dose–response manner. However, when the A-alone was treated with 2 and 5 µM of CMC2.24, only the higher concentration (5 µM) of CMC2.24 appeared to produce a more significant reduction in TNF-α concentrations (p < 0.05), although a trend of reduction was seen in the lower concentration (2 µM) of CMC2.24-treated group (p > 0.05). Finally, when the L + A group was treated with 2 and 5 µM of CMC2.24, a lesser effect on the “two-hit” group was observed (p > 0.05), even though there was still a trend of reduction in TNF-α concentrations.

### Effects of CMC2.24 on resolvins

**Figure 3:**
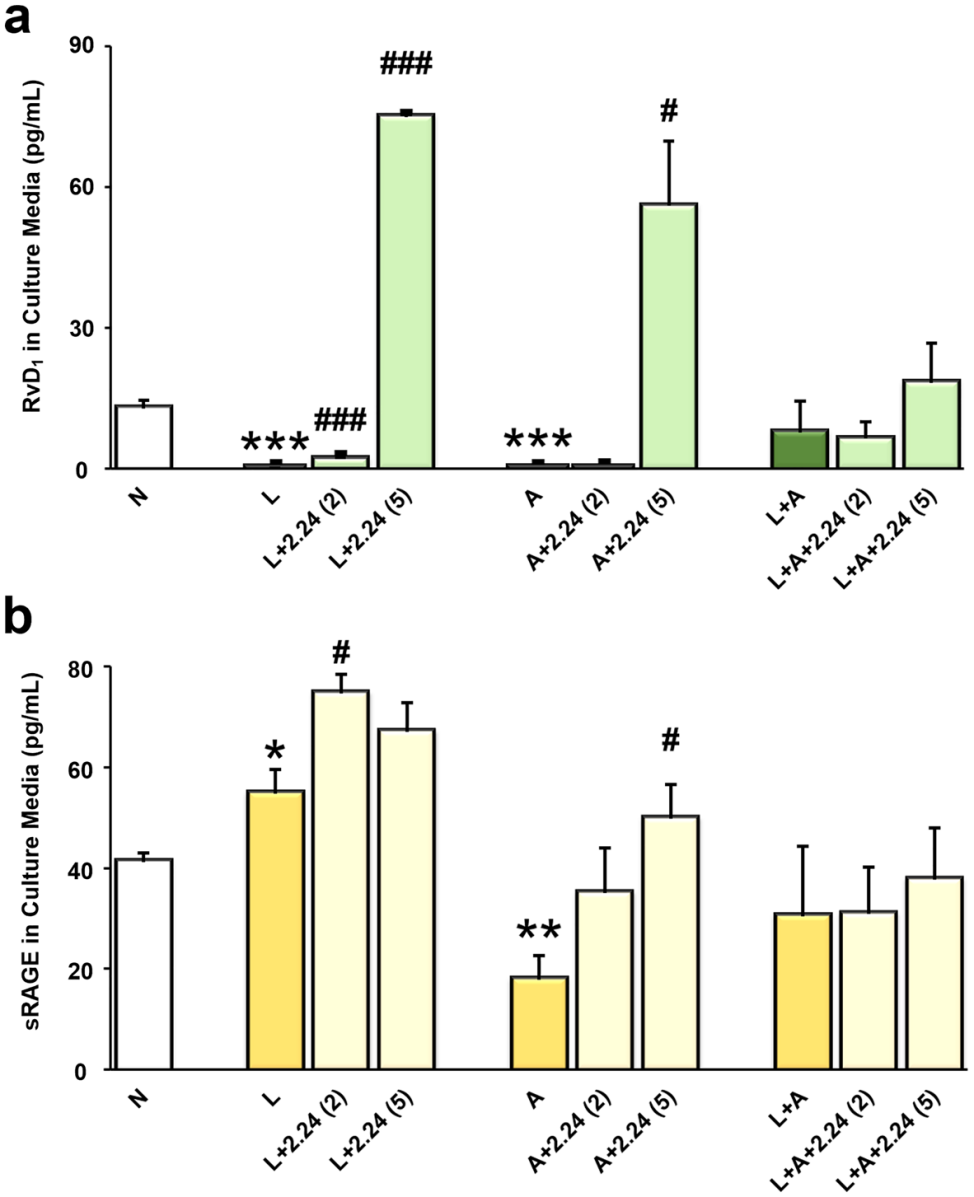
Effects of CMC2.24 on Resolvin D_1_ and soluble RAGE. The effects of CMC2.24 on the concentrations (pg/mL) of RvD_1_ (**a**) and sRAGE (**b**) in rat peritoneal Mφs challenged by LPS-alone, AGE-alone, or the “two-hit”, respectively; and treated with different concentrations (2 or 5 μM) of CMC2.24 at 18-h. N, the normal group. L, the LPS-alone challenged group. L + 2.24 (2 or 5), the low (2 μM) and high (5 μM) concentrations of CMC2.24-treated groups. A, the AGE-alone challenged group. A + 2.24 (2 or 5), the low (2 μM) and high (5 μM) concentrations of CMC2.24-treated groups. L + A, the “two-hit” challenged group. L + A + 2.24 (2 or 5), the low (2 μM) and high (5 μM) concentrations of CMC2.24-treated groups. Each value represents the mean (n = 3/group) ± the standard error. *p < 0.05, **p < 0.005, ***p < 0.001, values were compared between N and L/A/(L + A)-challenged at 18-h. ^#^p < 0.05, ^###^p < 0.001, values were compared between L/A/(L + A)-challenged and L/A/(L + A) + 2.24 (2 or 5)-treated groups at 18-h.

Regarding RvD_1_, as shown in Fig. 3a, the concentrations of this resolvin were significantly reduced to undetectable levels both in the L and A-alone groups (both p < 0.001), respectively, compared to the N group. When the L-alone was treated with 2 and 5 µM of CMC2.24, both concentrations significantly increased RvD_1_ concentrations (both p < 0.001) in a dose–response manner, compared to the L-alone. It should also be noted that the higher concentration (5 µM) of CMC2.24 treatment, but not the lower concentration (2 µM) tremendously increased RvD_1_ concentrations by eightfold above the L-alone challenged group and by 6-folds above normal (N) levels. Similarly, when the A-alone was treated with 2 and 5 µM of CMC2.24, the higher concentration (5 µM) of CMC2.24 significantly increased this resolvin by sixfold above the A-alone and 4-folds above N levels (both p < 0.05). However, these effects were less prominent in the “two-hit” group.

Concentrations of RvE_1_ were not statistically different among the normal, the challenged groups (L-, A-alone, and L + A), and the CMC2.24-treated groups. In addition, concentrations of LxA_4_ were undetectable in the rat peritoneal Mφs culture media (data not shown).

### Effects of CMC2.24 on sRAGE

Although LPS-challenge did not reduce the levels of sRAGE, the 2 µM of CMC2.24-treated group significantly raised the levels of sRAGE (p < 0.05); and a trend towards increase of the levels of this molecule was seen in the 5 µM of CMC2.24-treated group as well, both compared to the L-alone (Fig. 3b). Besides, the concentrations of sRAGE were dramatically reduced in the A-alone challenged group (p < 0.005) compared to the N group. Both concentrations of CMC2.24-treated groups increased the sRAGE concentrations. But the higher concentration (5 µM) of CMC2.24 seemed to be more potent than the lower one (2 µM), to bring the levels of sRAGE back to normal (p < 0.05), compared to the A-alone. In addition, the L + A group had a trend toward reduction in sRAGE levels, compared to the N group. However, there were fewer effects of CMC2.24 on sRAGE levels in the “two-hit” group (p > 0.05).

### Conversion of Mφs from M1 to M2 phenotype

#### LPS-challenged Mφs

**Figure 4:**
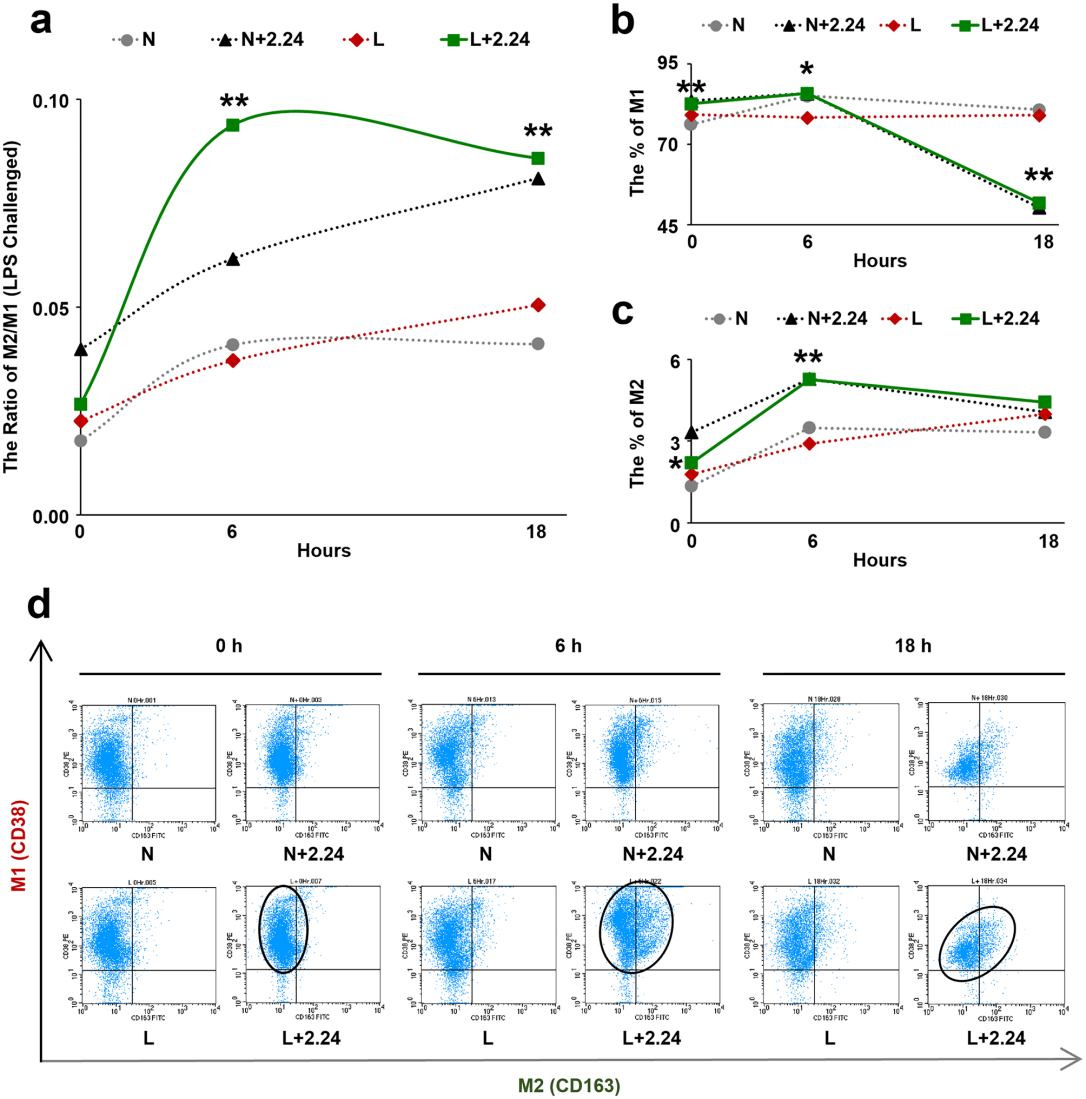
Conversion of LPS-challenged Mφs from M1 to M2 phenotype. At 0, 6, and 18 h, the effects of CMC2.24 on the ratio of M2 to M1 phenotype of L-challenged Mφs were indicated in (**a**) and analyzed by flow cytometry. The effects of CMC2.24 on each phenotype of L-challenged Mφs were indicated in (**b**) (for the percentage of M1), and (**c**) (for the percentage of M2), respectively. N, the normal group (grey line). N + 2.24, the CMC2.24 treated N group (black line). L, the LPS-alone challenged group (red line). L + 2.24, the CMC2.24-treated L group (green line). (**d**) Scatter diagrams of flow cytometry analysis showed the effects of CMC2.24 on the phenotype conversion of L-challenged Mφs, the “shift” from M1 to M2 phenotype after CMC2.24 treatment. Scatter diagrams of four groups (N, N + 2.24, L, L + 2.24) were present together at 0 h, 6 h, and 18 h. Each scatter diagram has 4 quadrants, UL: CD38^+^CD163^−^ (M1-only); UR: CD38^+^CD163^+^ (M1 and M2 mixed); LL: CD38^−^CD163^−^ (M0); LR: CD38^−^CD163^+^ (M2-only). Each value represents the mean (n = 3/group) ± the standard error. *p < 0.05, **p < 0.005, values were compared between L-challenged and L + 2.24-treated groups at 0, 6, and 18 h.

At 0, 6, and 18 h, the ratio of M2/M1 was indicated in Fig. 4a. With 6 to 18-h treatment of CMC2.24, this ratio was dramatically increased in the L + 2.24 group, and was significantly higher than the L-alone group (p < 0.005). Interestingly, the ratio of M2/M1 in N + 2.24 was also higher at these time points compared to the untreated (N and L-alone) groups. Thus, treatment with CMC2.24 appeared to “prime” the normal monocyte/macrophage to develop to the M2 phenotype. In the absence of CMC2.24, the ratio of M2/M1 in the L-alone and N groups was maintained at low levels at each time point (Fig. 4a). Consistent with the ratio change of M2/M1, the percentage of M1 population was dramatically decreased in the L + 2.24 group at 18 h, compared to the L-alone (p < 0.005) (Fig. 4b). However, the percentage of M2 population was significantly increased in the CMC2.24-treated group at 6 h, compared to the L-alone (p < 0.005) (Fig. 4c).

These observations were further confirmed by the scatter diagrams of flow cytometry analysis (Fig. 4d). In the L + 2.24 group, we observed a population of Mφs that was gradually “shifting” from M1 towards M2 phenotype during the time. This “shifting” population was indicated in the CMC2.24-treated Mφs (L + 2.24; N + 2.24). However, the population of Mφs remained unchangeable in the N and L-alone groups.

#### AGE-challenged Mφs

**Figure 5:**
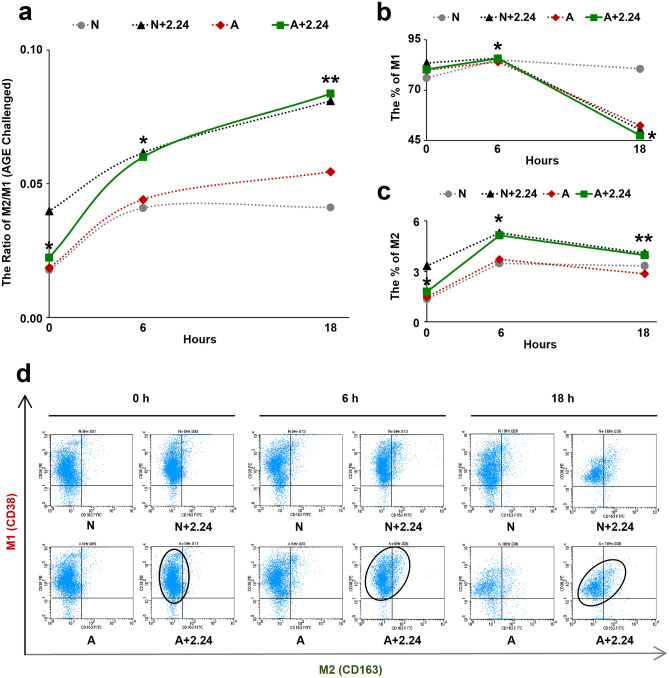
Conversion of AGE-challenged Mφs from M1 to M2 phenotype. At 0, 6, and 18 h, the effects of CMC2.24 on the ratio of M2 to M1 phenotype of A-challenged Mφs were indicated in (**a**) and analyzed by flow cytometry. The effects of CMC2.24 on each phenotype of A-challenged Mφs were indicated in (**b**) (for the percentage of M1), and (**c**) (for the percentage of M2), respectively. N, the normal group (grey line). N + 2.24, the CMC2.24 treated N group (black line). A, the AGE-alone challenged group (red line). A + 2.24, the CMC2.24-treated A group (green line). (**d**) Scatter diagrams of flow cytometry analysis showed the effects of CMC2.24 on the phenotype conversion of A-challenged Mφs, the “shift” from M1 to M2 phenotype after CMC2.24 treatment. Scatter diagrams of four groups (N, N + 2.24, A, A + 2.24) were present together at 0 h, 6 h, and 18 h. Each scatter diagram has 4 quadrants, UL: CD38^+^CD163^−^ (M1-only); UR: CD38^+^CD163^+^ (M1 and M2 mixed); LL: CD38^−^CD163^−^ (M0); LR: CD38^−^CD163^+^ (M2-only). Each value represents the mean (n = 3/group) ± the standard error. *p < 0.05, **p < 0.005, values were compared between A-challenged and A + 2.24-treated groups at 0, 6, and 18 h.

A similar trend was observed in the AGE-challenged group (Fig. 5a). At 0, 6, and 18 h, the ratio of M2/M1 in the A + 2.24-treated group was markedly increased after 6 to 18-h treatment with CMC2.24 (p < 0.05), and was significantly higher than the A-alone group (p < 0.005). Consistent with the previous observation, the ratio of M2/M1 in N + 2.24 was also higher at these time points, compared to the untreated (N and A-alone) groups. Again, treatment with CMC2.24 “primed” the normal monocyte/macrophage to convert to the M2 phenotype under these conditions. However, in the absence of CMC2.24, the ratio of M2/M1 in the A-alone and N groups was maintained at low levels at each time point (Fig. 5a). This can be indicated in Fig. 5b,c, respectively. The M1 population appeared to be slightly decreased in the A + 2.24 group at 18 h, compared to the A-alone (p < 0.05) (Fig. 5b). However, the M2 population was significantly increased in the A + 2.24 group at 6 and 18 h, compared to the A-alone (p < 0.05; p < 0.005) (Fig. 5c).

Again, these observations were further confirmed by the scatter diagrams of flow cytometry analysis (Fig. 5d). A population of Mφs was gradually “shifting” from M1 towards M2 phenotype in the A + 2.24 group during the time. This “shifting” population was indicated in the CMC2.24-treated Mφs (A + 2.24; N + 2.24). However, the population of Mφs remained unchangeable in the N and A-alone groups.

## Discussion

In this study, we demonstrated the current view on the pathogenesis of diabetes-associated periodontitis, which includes a series of dysregulated host inflammatory/collagenolytic responses. HMT is a novel therapeutic strategy to resolve uncontrolled and unresolved inflammation, which could be an adjunct to conventional treatment of diabetes-associated periodontitis.

A NON-antimicrobial formulation of doxycycline (NAD), as the first generation of HMT agents developed by our group, has been and still is the only governmentally-approved agent for clinically treating periodontitis in humans^52^. A second generation of HMT agents, namely chemically-modified tetracyclines (CMTs, especially CMT-3), has also shown evidence of safety and efficacy in cell and tissue culture, in vivo (rat models), and in clinical studies^51–54^. Many recent studies indicate that curcumin can modulate macrophage polarization through its anti-inflammatory effects^55, 56^. As the latest generation of HMT agents, CMC2.24 is a phenylaminocarbonyl curcumin which has a triketonic structure at its C-4 position, resulting in higher bioactivity, better solubility, and greater zinc-binding capability than natural curcumin, as well as no evidence of toxicity even at high doses^36, 57, 58^. The novelty of CMC2.24 has also been tested in different models and conditions as we described previously^28–30, 32–34^. For example, we examined the signaling and transcriptional factors in two in vivo models of diseases (diabetes and periodontitis)^28, 29, 31, 32^. The results demonstrated that CMC2.24 significantly reduced TLR-2 and p38 MAPK expressions involved in the inflammatory signaling cascade in a dog model of natural periodontitis; and inhibited NF-κB activation and inflammatory bone loss in murine models of LPS-induced experimental periodontitis and diabetes-associated natural periodontitis.

More importantly, our group also has compared the effects of the oral administration of natural curcumin and a chemically-modified curcumin (CMC2.24) on osteoclast-mediated bone resorption, apoptosis, and inflammation in a rat model of experimental periodontitis^59^. We found that CMC2.24 and curcumin inhibit inflammation by different mechanisms, CMC2.24 was capable of reducing alveolar bone resorption in the LPS-induced model of periodontitis. In addition, we did an in vitro study regarding the inhibitory concentration at 50% (IC50) of CMC2.24 and natural curcumin. It demonstrated that CMC2.24 exhibited inhibitory IC50 values in vitro, ranging from 2–8 μM against two collagenases (MMP-8 and MMP-13), two gelatinases (MMP-2 and MMP-9), MMP-3, MMP-7 and MMP-12, which were much lower than curcumin, ranging from 3 to 52 μM^57^. Therefore, CMC2.24 exhibited stronger potency and efficacy than natural curcumin on MMPs inhibition.

**Figure 6:**
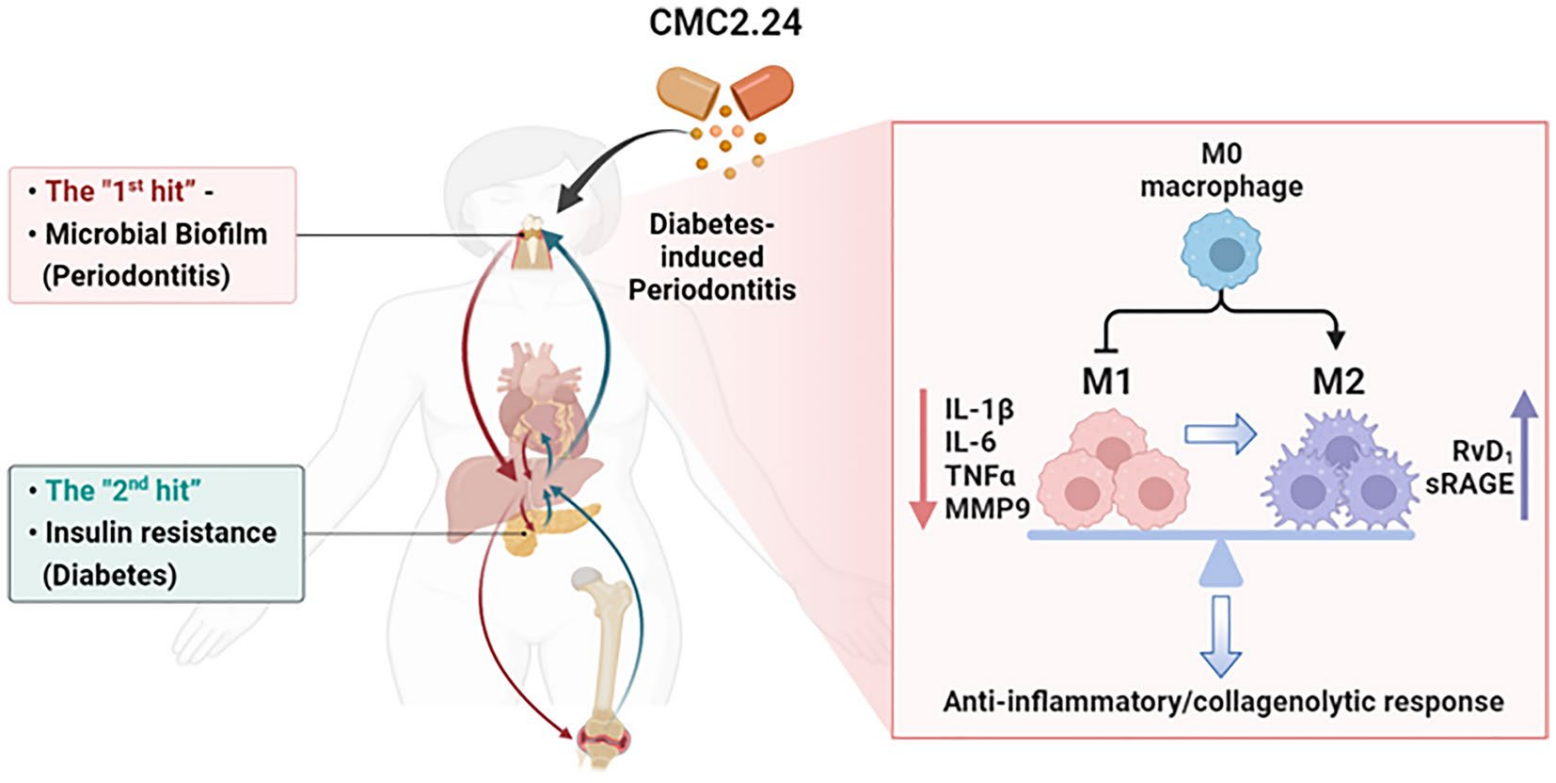
A scheme to summarize the pleiotropic mechanisms of the novel chemically-modified curcumin 2.24 on resolving inflammation in a “two-hit” model. This “two-hit” model demonstrates a bi-directional manner between diabetes (or other systemic diseases) and periodontitis. Treatment with CMC2.24 can inhibit inflammatory and tissue-destructive mediators including cytokines and MMPs; upregulate resolvin-enhancing (RvD1) activity; and promote the expression of sRAGE; as well as contribute to macrophage polarization from the M1 towards M2 phenotype.

For these reasons, currently, we focused on the multiple mechanisms of efficacy of CMC2.24. This *ex-vivo* study provided insights that CMC2.24 can restore the balance between the pro-inflammatory and the anti-inflammatory/pro-resolving responses, (a) by decreasing the production of pro-inflammatory mediators (IL-1β, IL-6, and TNF-α), as well as the collagenolytic MMP (MMP-9); and (b), by also dramatically increasing RvD_1_ levels, as well as sRAGE levels. Many studies showed that the cytokines, like IL-1β, TNF-α, and IL-6 were involved in MMPs upregulation^60^, and connective tissue breakdown, including bone resorption^17^. Therefore, our results indicate that CMC2.24 is a potent regulator with additional mechanisms to resolve inflammation and restore homeostasis. The pleiotropic mechanisms of this novel compound on resolving inflammation have been summarized in Fig. 6.

RvD_1_ is a well-characterized D-series resolvin that is generated in situ from DHA by two sequential lipoxygenation steps^61^. Many researchers have found that RvD_1_ is important in (a) ameliorating LPS-induced inflammatory diseases^62^; (b) improving insulin sensitivity by resolving the chronic inflammation that is associated with diabetes and obesity^63^; and importantly, (c) activating the anti-inflammatory response, partially by the conversion of Mφs from classically-activated (M1 phenotype) to alternatively activated (M2 phenotype) cells^14^. This “shift” accelerates the resolution of persistent inflammation at the cellular level. And our study identified that RvD_1_, not the other resolvins or lipoxins such as RvE_1_ and LxA_4_, is the main target for the resolvin-like activity of CMC2.24. This did provide the additional mechanisms that help explain its potent efficacy in the resolution of tissue-destructive effects of chronic inflammation.

Besides, the role of AGE-RAGE interaction in promoting chronic inflammatory diseases has been increasingly recognized. AGE is formed when reducing sugars nonenzymatically bind to proteins or lipids, a process that is enhanced by hyperglycemic and hyperlipidemic environments of numerous metabolic disorders including diabetes and its complications (e.g., periodontitis)^9^. sRAGE, a decoy receptor for AGE, is associated with diabetes when a lower level of this soluble receptor which present in the circulation. In this study, AGE-challenged Mφs showed a decrease in the levels of sRAGE. But CMC2.24-treated Mφs significantly increased the levels of sRAGE and brought them back to normal. This indicated an additional mechanism that CMC2.24 resolve inflammation through AGE-RAGE/sRAGE signaling.

Moreover, another mechanism of CMC2.24’s pleiotropic efficacy is indicated by the conversion of the M1 pro-inflammatory phenotype to the M2 pro-resolving phenotype. In terms of both phenotype and function, Mφs have remarkable heterogeneity^64^, reflected in their specialization and also the marked changes in their activity and gene expression when facing challenges in the microenvironment. Our study demonstrated that the CMC2.24-treated Mφs significantly increased the ratio of M2/M1, and enhanced the conversion of Mφs from the M1 to M2 phenotype. This effect appeared to be attributed primarily due to the suppression of the M1 phenotype population; and due to the early promotion of the M2 phenotype population. As we observed, at 18 h, the challenge with either LPS or AGE and treatment with CMC2.24 can dramatically suppress the M1 phenotype as indicated by a lower expression of the M1 cell surface marker, CD38, compared to the non-treated or challenge-only groups. In contrast, the M2 phenotype population under the same conditions was markedly increased at 6 h after CMC2.24 therapy. This early effect can be lasting for 18 h as made evident by a high expression of the M2 cell-surface marker, CD163. The scatter diagram confirmed our hypothesis that CMC2.24 produced a “shifting” of Mφs from the M1 towards the M2 phenotype using flow cytometry (Figs. 4d, 5d). Therefore, CMC2.24 appears to be a highly potent inhibitor of the M1 phenotype (probably related to the inhibition of the M1 macrophage differentiation or maturation, or both); and a promoter of the pro-resolving M2 macrophage, thus acting like a “switch”.

## Conclusion

Taken together, these results continue to expand the pleiotropic mechanisms of the novel chemically-modified curcumin 2.24, by (a) inhibiting inflammatory and tissue-destructive mediators including cytokines and MMPs; (b) upregulating resolvin-enhancing (RvD_1_) activity; and (c) promoting the expression of sRAGE; as well as (d) contributing to macrophage polarization from the M1 towards M2 phenotype (Fig. 6). Therefore, CMC2.24 can be a promising drug in treating diabetes-associated periodontitis. And the challenge for the future will be to demonstrate the safety and efficacy of this pleiotropic compound for the treatment of human chronic inflammatory diseases including diabetes, periodontitis, and others.

### Supplementary Information

Supplementary Information.

## Data Availability

Data supporting the findings of the current study are available from the corresponding author upon reasonable request.

## Abbreviations

AGE/A: Advanced-glycation-end products
CFPFs: Cell-free peritoneal fractions
CMC2.24: Chemically-modified curcumin 2.24
EDTA: Ethylenediaminetetraacetic acid
ELISA: Enzyme-linked immunosorbent assay
HMT: Host modulation therapy
IL-1β: Interleukin-1 beta
IL-6: Interleukin-6
LPS/L: Lipopolysaccharide
LxA_4_: Lipoxin A_4_
Mφs: Macrophages
MMPs (e.g., MMP-2 or -9): Matrix metalloproteinases
P.g.: Porphyromonas gingivalis
PMNs: Polymorphonuclear leukocytes
PW: Peritoneal washes
RAGE: Receptors for AGE
RvD_1_: Resolvins D_1_
RvE_1_: Resolvins E_1_
sRAGE: soluble receptor for AGE
TNF-α: Tumor necrosis factor-alpha
