# Supplementary figures and images for: A novel modified-curcumin 2.24 resolves inflammation by promoting M2 macrophage polarization
Source: Sci Rep. 2023 Sep 19;13:15513. doi: 10.1038/s41598-023-42848-x (PMC10509274; doi:10.1038/s41598-023-42848-x)

**a**

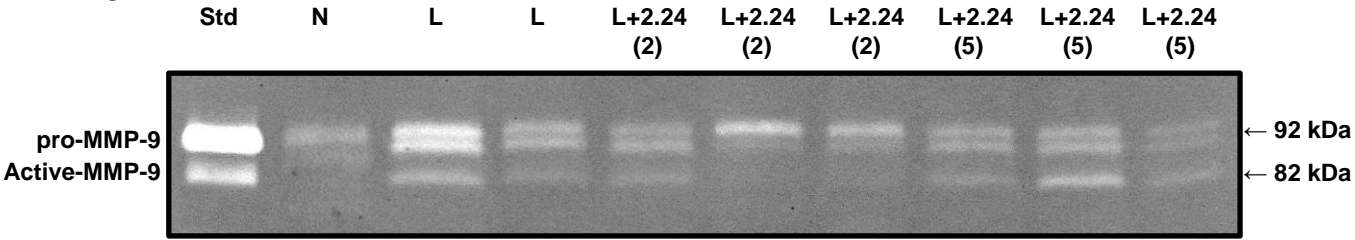

**b**

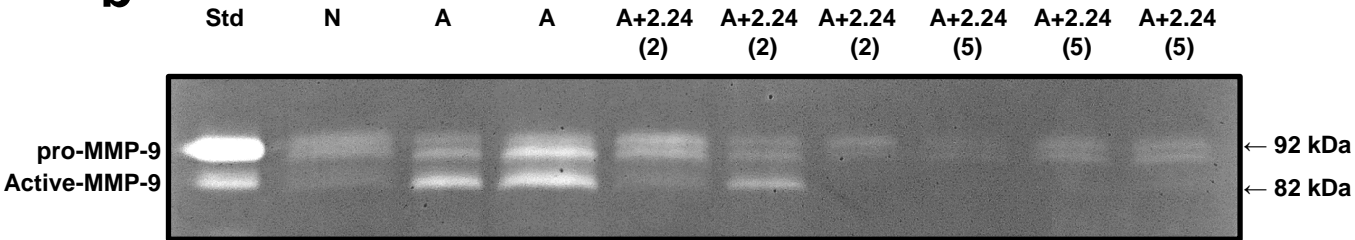

**c**

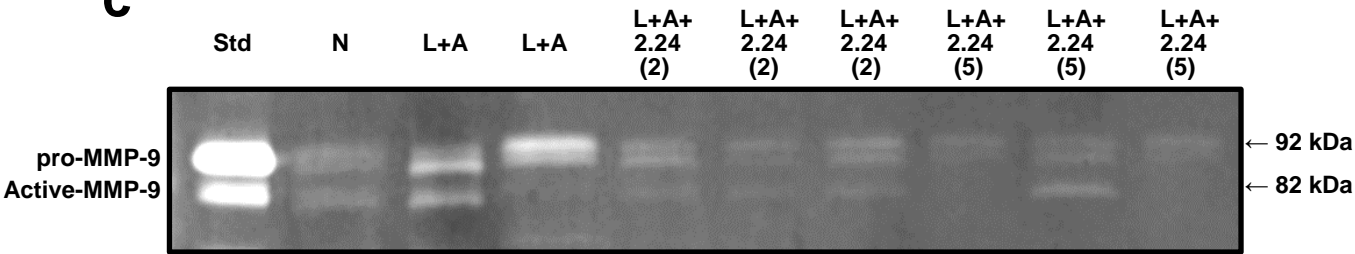

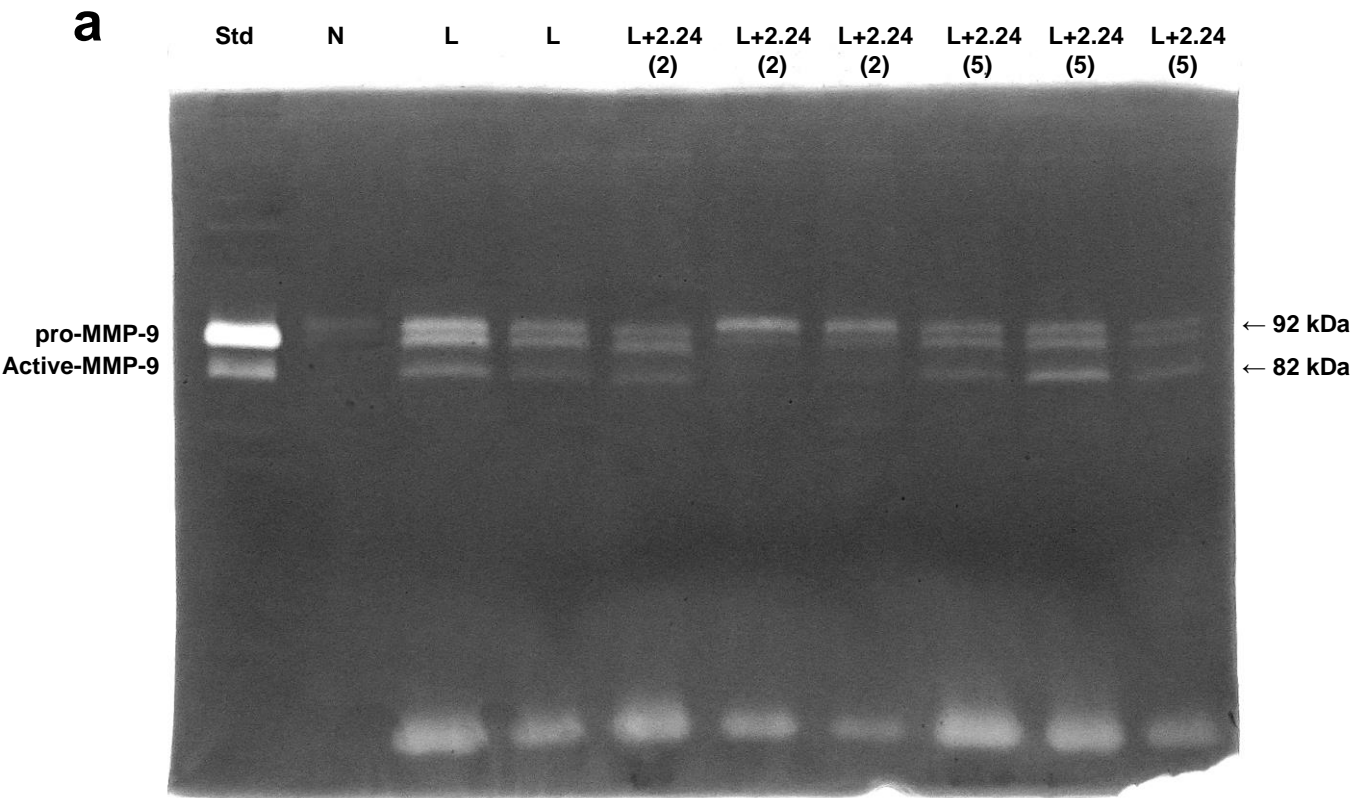

**S 3.**

**b**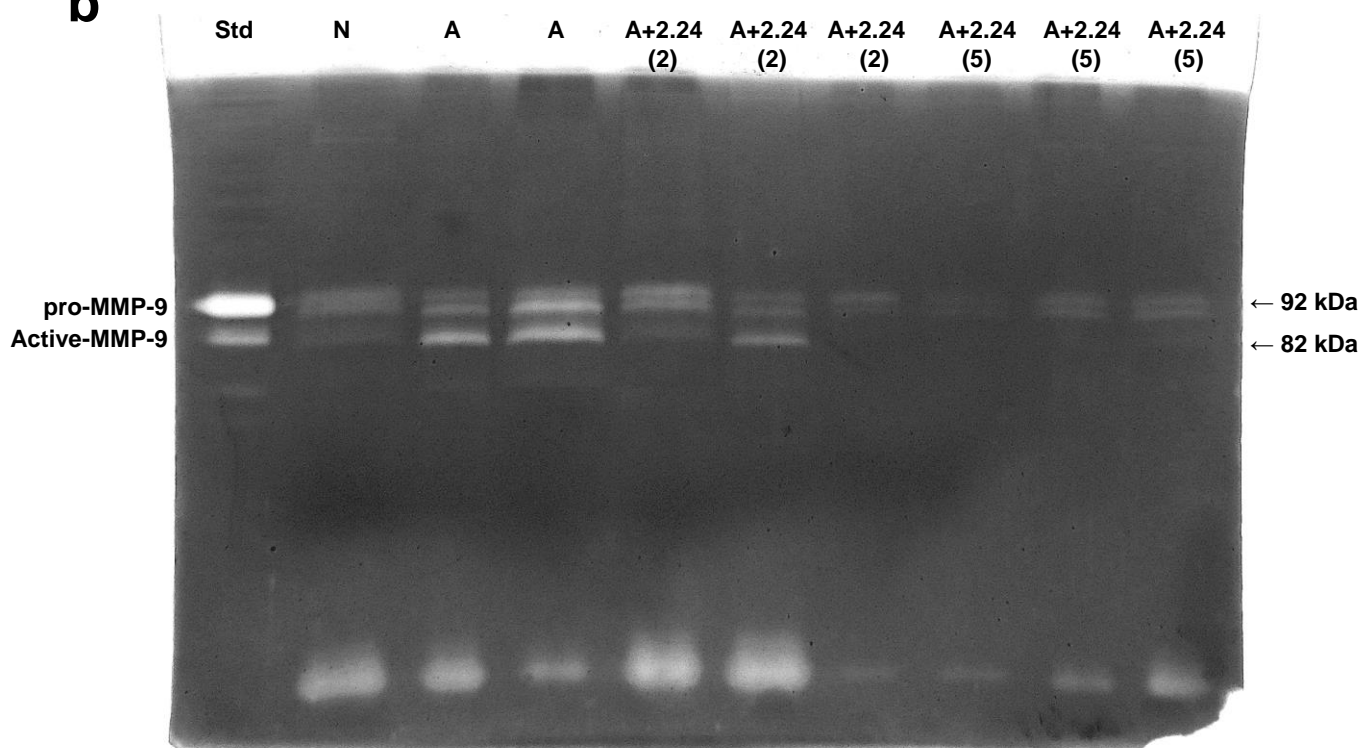

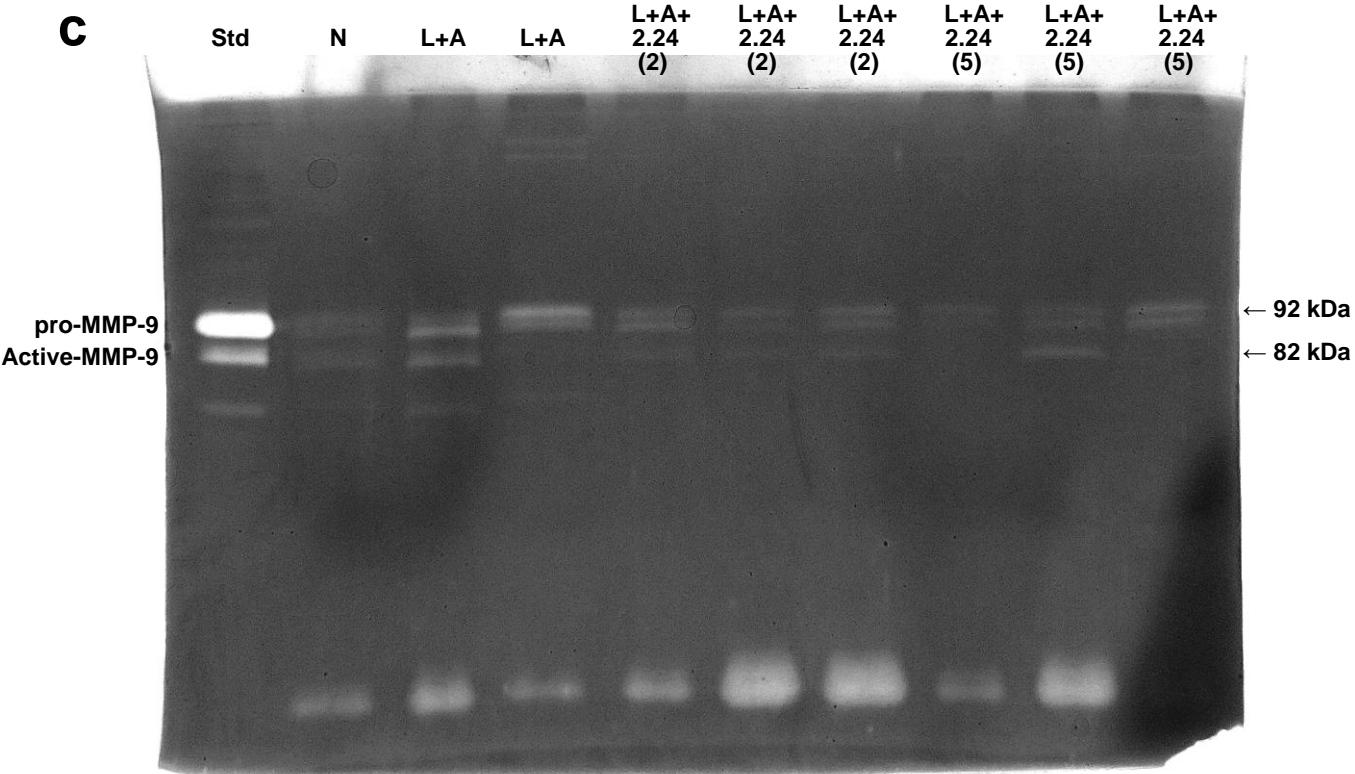

Supplement: Supplementary file 1 — Supplementary Information. [file 41598_2023_42848_MOESM1_ESM.pdf]
